# Editorial: Challenges of maternal and child health after the COVID-19 pandemic

**DOI:** 10.3389/fpubh.2023.1224093

**Published:** 2023-06-08

**Authors:** Kimiyo Kikuchi, Keiko Nanishi, Siyan Yi, Junko Yasuoka

**Affiliations:** ^1^Department of Health Sciences, Faculty of Medical Sciences, Kyushu University, Fukuoka, Japan; ^2^Office of International Academic Affairs, Graduate School of Medicine and Faculty of Medicine, The University of Tokyo, Tokyo, Japan; ^3^Saw Swee Hock School of Public Health, National University of Singapore and National University Health System, Singapore, Singapore; ^4^KHANA Center for Population Health Research, Phnom Penh, Cambodia; ^5^Center for Global Health Research, Touro University California, Vallejo, CA, United States; ^6^Center for Infectious Disease Epidemiology and Prevention Research, Tokyo University of Agriculture and Technology, Tokyo, Japan

**Keywords:** COVID-19, editorial, maternal health, child health, mental health

The COVID-19 pandemic has impacted people's access to and quality of care (1). In several countries, maternal and child health services, including perinatal care, immunization, and facility delivery, declined during the pandemic (2), and emergency care faced the risk of interruption. Accordingly, innovative solutions are required to address these challenges (3). Further research is warranted to determine how these restrictions can affect the health of mothers and children. Moreover, understanding these constraints may prevent further maternal and child healthcare disruptions in future public health emergencies. Thus, we initiated this Research Topic to highlight the status, impact, and solutions of maternal and child healthcare access and quality challenges during and after the pandemic.

Chao et al. systematically reviewed 78 articles to rank COVID-19-related symptoms, such as cough, fever, myalgia, headache, and dyspnea, among pregnant women. The focus of the study was relevant, given that pregnant women are less likely to manifest COVID-19 symptoms and more likely to warrant COVID-19-related intensive care unit admission than non-pregnant women. Therefore, this symptom ranking list may aid clinical screening to determine COVID-19 infection among pregnant women. Regarding COVID-19's impact on child health, Puspitarani et al. reported on adverse events following immunization (AEFI) in children. According to 1,093 parents of children (aged 6–11 years) in Yogyakarta, Indonesia, who received first and second doses of inactivated severe acute respiratory syndrome coronavirus 2 (SARS-CoV-2) vaccine, mild AEFI was associated with factors, including previous AEFI experience and receipt of other vaccines containing the same adjuvant as CoronaVac within 1 month.

Cena et al. reported on access to maternal and perinatal healthcare services in Italy during the pandemic. Of the 77 public and private maternity and perinatal centers, 70% reported that the first wave of the pandemic had adversely affected the functioning of one or more aspects of perinatal services, and 23% were understaffed. These findings indicate that healthcare systems are poorly prepared to handle health services during pandemic emergencies. Carter et al. assessed the impact of COVID-19 on the reproductive, maternal, and newborn health services in Ethiopia. According to the Performance Monitoring for Action-Ethiopia data, the health service provision was minimally disrupted during the initial months of the pandemic, although the number of stillbirths increased in the COVID-19 cohort. Based on distinct findings documented in Italy and Ethiopia, maternal and child healthcare access and consequences differ depending on the medical resource background of the country.

During the COVID-19 pandemic, family care for patients was affected by changes in family visitation and chaperone protocols during hospitalization. In their narrative review, Lessa et al. discussed the negative impact of the pandemic on patient- and family-centered care (PFCC) in the pediatric intensive care unit, including family support and communication. The authors highlighted several strategies used to maintain PFCC and achieve the minimal goal of humanized care during the pandemic.

Several studies have explored the health status of children and their caregivers during this pandemic. Ma et al. reported the prevalence of stunting among children <3 years of age in Longgang, China, and its risk factors during the pandemic. Among 118,404 kindergarten children, the authors identified distal, proximal, and intermediate factors, suggesting the need to strengthen feeding behaviors and healthy lifestyles to prevent stunting in children during a pandemic. Upadyaya et al. identified distinct homogeneous profiles of parents who experienced burnout in Finland during the pandemic. Considering 1,314 parents from the Helsinki Metropolitan area, those whose children faced challenges tended to experience high burnout profiles. Wang et al. explored the association between parental wellbeing and child mental health problems during the pandemic. According to their analyses of data from a population-based survey of parents of children aged 3–6 years across mainland China, higher parental mental health wellbeing, measured by World Health Organization-Five Wellbeing Index, was associated with poor child mental health, noting that harsh parenting and child sleep issues mediated the association. Kokkinaki and Hatzidaki reviewed factors that negatively affected perinatal mental health during pandemic-related restrictions and revealed that maternal emotional wellbeing adversely affected infant development. The authors also highlighted the need to integrate evidence-based promotion of family mental health into prenatal and postnatal care to facilitate patient care. In Germany, Gulde et al. conducted a path model study among 73 mothers recruited shortly after birth at the University Hospital of Ulm between 2013 to 2015 and surveyed during the COVID-19 pandemic. Maternal attachment representation appeared unstable and lacked coping strategies, given the various pandemic-related limitations (Gulde et al.). This may lead to harmful parental behaviors and ultimately affect children's mental health. Fielding-Gebhardt et al. conducted a study at the University of Kansas, United States, regarding the mental wellbeing of 37 mothers of children with fragile X syndrome during the COVID-19 pandemic, who are themselves carriers of the FMR1 gene premutation (Fielding-Gebhardt et al.). The mothers experienced trouble adapting and coping with the circumstances during the pandemic, although positive adaptations, such as increasing feelings of family togetherness, were also observed.

Several factors are associated with perinatal mental health among mothers. According to a review by Bottemanne et al., fear of being infected/infecting others and uncertainty regarding the effect of the virus on fetuses and infants might affect mothers' mental health. As a potential solution to mental health issues, Liu et al. reported the effectiveness of a mental health program implemented in Massachusetts, the United States. Helping Us Grow Stronger (HUGS/Abrazos) is an emergency assistance program supporting vulnerable patients, including pregnant women and children. The program integrated and streamlined social and behavioral health support, which served as a buffer to protect pregnant women and families with young children and foster resilience.

Sharing correct COVID-19 information with family members could be related to family wellbeing, including perceived family health, happiness, and harmony. Using the Jockey Club SMART Family-Link Project in Hong Kong, Wong et al. conducted a study among 4,891 adults. The authors revealed that family wellbeing was associated with the confirmation of correct information, followed by sharing with families. Accordingly, public healthcare professionals should encourage verifying and forwarding COVID-19-related details to family members to ensure family communication and wellbeing (Wong et al.).

In conclusion, this special issue examines diverse maternal and child health challenges introduced by the COVID-19 pandemic. The subtopics included (a) COVID-19-related symptoms in pregnant women, (b) vaccine-induced adverse events in children, (c) access to and implementation of perinatal care varying by country and time context, (d) challenges faced by families of pediatric patients owing to limitations on visitations, and (e) mental health of mothers and children and the support program. The present editorial discusses the impact of the COVID-19 pandemic on mothers and children, including both direct and indirect causes, such as long-term behavioral restrictions, considering mental health and parent-child and family relationships. These studies provide essential information to circumvent critical situations during future pandemics.

## Author contributions

KK, KN, SY, and JY were the associate editors. KK was the editorial author. All authors contributed to the article and approved the submitted version.
